# Impact of severe left ventricular dysfunction on in-hospital and mid-term outcomes of Chinese patients undergoing first isolated off-pump coronary artery bypass grafting

**DOI:** 10.1186/s13019-017-0651-z

**Published:** 2017-10-10

**Authors:** Qiang Ji, Li Min Xia, Yun Qing Shi, Run Hua Ma, Jin Qiang Shen, Wen Jun Ding, Chun Sheng Wang

**Affiliations:** 1 Department of Cardiovascular Surgery of Zhongshan Hospital Fudan University, Shanghai, 180 Fenglin Rd, Shanghai, 200032 People’s Republic of China; 2 Shanghai Institute of Cardiovascular Disease, 1609 Xietu Road, Shanghai, 200032 People’s Republic of China

**Keywords:** Coronary artery bypass grafting, off-pump, Severe left ventricular dysfunction, In-hospital mortality, Major postoperative morbidity, Mid-term survival

## Abstract

**Background:**

Few studies focused on evaluating the impacts of preoperative severe left ventricular dysfunction on clinical outcomes of patients undergoing off-pump coronary artery bypass grafting surgery (OPCAB). This single center retrospective study aimed to evaluate the impacts of severe left ventricular dysfunction on in-hospital and mid-term clinical outcomes of Chinese patients undergoing first, scheduled, and isolated OPCAB surgery.

**Methods:**

From January 2010 to December 2014, 2032 eligible patients were included in this study and were divided into 3 groups: a severe group (patients with preoperative left ventricular ejection fraction (LVEF) of ≤35%, *n* = 128), an impaired group (patients with preoperative LVEF of 36-50%, *n* = 680), and a normal group (patients with preoperative LVEF of >50%, *n* = 1224). In-hospital and follow-up clinical outcomes were investigated and compared.

**Results:**

Patients in the severe group compared to the other 2 groups had higher in-hospital mortality and higher incidences of low cardiac output and prolonged ventilation. Kaplan-Meier curves showed a similar cumulative follow-up survival between the severe group and the impaired group (χ^2^ = 1.980, Log-rank *p* = 0.159) and between the severe group and the normal group (χ^2^ = 2.701, Log-rank *p* = 0.102). Multivariate *Cox* regression indicated that grouping was not a significant variable related to mid-term all-cause mortality. No significant difference was found in the rate of repeat revascularization between the severe group (2.4%) and the other 2 groups.

**Conclusions:**

Patients with preoperative LVEF of ≤35% compared to preoperative LVEF of >35% increased the risk of in-hospital death and incidences of postoperative low cardiac output and prolonged ventilation, but shared similar mid-term all-cause mortality and repeat revascularization after OPCAB surgery.

## Background

Surgical revascularization for patients with coronary artery disease and severe left ventricular dysfunction compared to percutaneous coronary intervention prevents further myocardial damage, preserves the remaining myocardium, and induces the recovery of systolic function in ischemic left ventricular myocardial segments, associating with improved survival and lower rate of repeat revascularization [1, 2]. A large number of previous studies [1–14] focused on evaluating the impacts of preoperative severe left ventricular dysfunction (either left ventricular ejection fraction (LVEF) of ≤35% or LVEF of ≤30%) on in-hospital and follow-up outcomes of patients with coronary artery disease undergoing surgical revascularization. However, the vast majority of patients in previous reports received on-pump coronary artery bypass grafting (CABG) surgery with or without concomitant surgery. Few studies focused on evaluating the impacts of preoperative severe left ventricular dysfunction on in-hospital and follow-up outcomes of patients with coronary artery disease after receiving isolated off-pump CABG surgery (OPCAB). Recently, a meta-analysis of 4119 patients with coronary artery disease and preoperative severe left ventricular dysfunction (LVEF of ≤35%) from 26 observational clinical studies reported surgical mortality after on-pump CABG and OPCAB surgery and 5-year actuarial survival rate after on-pump CABG surgery, but it lacked 5-year actuarial survival rate after OPCAB surgery [15]. Additionally, coronary artery disease has a significant genetic heterogeneity between Chinese and Westerners. Therefore, it was necessary to evaluate the impacts of severe left ventricular dysfunction on in-hospital and follow-up clinical outcomes of Chinese patients with coronary artery disease who received surgical revascularization.

This single center retrospective study aimed to evaluate the impacts of preoperative severe left ventricular dysfunction compared to preoperative mild to moderate left ventricular dysfunction and preoperative normal left ventricular function on in-hospital and follow-up clinical outcomes through reviewing and following up 2032 patients with coronary artery disease who received first, scheduled, and isolated OPCAB surgery.

## Methods

### Evaluation of left ventricular function

Evaluation of left ventricular function was conducted by transthoracic echocardiography preoperatively, 1-2 weeks after surgical revascularization, and again during follow-up. Echocardiographic images were obtained from the parasternal, apical and subcostal windows for the evaluation of left ventricular function using commercially available systems. The LVEF was calculated from left ventricular end-diastolic volume (LVEDV) and left ventricular end-systolic volume (LVESV) estimates, using the following formula:

LVEF = (LVEDV - LVESV)/ LVEDV.

Left ventricular volume estimates may be derived from two-dimensional or three-dimensional echocardiography. The biplane method of disks (modified *Simpson’s* rule) is the currently recommended two-dimensional method to assess LVEF by consensus of this committee. For patients with good image quality, three-dimensional-echocardiography-based LVEF measurements are accurate and reproducible, and should be used when available and feasible [16].

In this study, severe left ventricular dysfunction, mild to moderate left ventricular dysfunction, and normal left ventricular function were defined as the values of LVEF of 35% or less, 36-50%, and more than 50%, respectively.

### Study protocols

Chinese patients with coronary artery disease and complete information from medical records who received first, scheduled, and isolated OPCAB surgery in our medical center from January 2010 to December 2014 were included into this study. Patients with preoperative concomitant malignant tumor were excluded.

All enrolled patients received OPCAB surgery (For detailed OPCAB technique see the literature [17]). In our medical center, statin medication, aspirin, and clopidogrel were routinely prescribed to all of the patients starting from postoperative day 1 or 2. Statin medication and aspirin were continued indefinitely, whereas clopidogrel was discontinued after 1 year [18–20].

All enrolled patients were divided into 3 groups: a severe group (patients with preoperative LVEF of 35% or less), an impaired group (patients with preoperative LVEF of 36-50%), and a normal group (patients with preoperative LVEF of more than 50%). Baseline and procedure characteristics and in-hospital clinical outcomes were reviewed, and mid-term clinical outcomes were followed up.

### Clinical data

The following baseline demographic and clinical variables were considered: age, older age (age > 65 years), gender (male/female), recent smoking (smoking within 4 weeks of surgery), diabetes, hypertension, hyperlipidemia, chronic obstructive pulmonary disease, renal dysfunction (creatinine more than 2.5 mg/dl or requiring dialysis), history of cerebro-vascular disease, history of percutaneous cardiac intervention, congestive heart failure (New York Heart Association grade III and IV), recent myocardial infarction (evidence of myocardial infarction within the last 30 days before surgery), coronary artery lesion (double-vessel or triple-vessel, left main coronary artery disease), and enlarged left ventricle (left ventricular endo-diastolic diameter (LVEDD) of more than 65 mm). Intra-operative variables of interest included the number of distal anastomoses, use of left internal mammary artery and saphenous vein graft as well as radial artery, urgent switch from off-pump to on-pump CABG, and prophylactic use of intra-aortic balloon pump (IABP).

The in-hospital clinical outcomes included in-hospital mortality and major postoperative morbidity including low cardiac output, new onset of myocardial infarction, re-operation for bleeding, prolonged ventilation, stroke, acute renal failure requiring hemodialysis, and deep sternal wound infection. In-hospital mortality was defined as death that occurred during the same hospitalization or within 30 days of operation. Postoperative low cardiac output was defined as the requirement for IABP for inability to discontinue cardiopulmonary or for longer than 30 min after the patient was returned to intensive care unit to maintain the systolic blood pressure > 90 mmHg and the cardiac index >2.2 l/min/m^2^. Myocardial infarction associated with CABG was arbitrarily defined by elevation of cardiac biomarker values >10 × 99th percentile upper reference limit in patients with normal baseline cardiac troponin values (≤ 99th percentile upper reference limit). In addition, myocardial infarction associated with CABG was defined by either (i) new pathological Q waves or new left bundle branch block, or (ii) angiographic documented new graft or new native coronary artery occlusion, or (iii) imaging evidence of new loss of viable myocardium or new regional wall motion abnormality. Prolonged ventilation was defined as the duration of mechanical ventilation of more than 48 h. Stroke was defined as any new temporary or permanent focal or global neurological deficit, in accordance with the published guidelines, within 30 days from operation or later than 30 days if still in hospital. And the incidence of deep sternal wound infection (bone related; any drainage of purulent material from the sternotomy wound and instability of the sternum), application of IABP on an “as needed” basis (patients were installed with an IABP when developing low cardiac output after CABG surgery), and the length of intensive care unit (ICU) stay were also recorded.

The follow-up outcomes among patients discharged alive from hospital were all-cause mortality and repeat revascularization. All-cause mortality rather than cardiac mortality was chosen because it is the most robust and unbiased index which exempted us from misreading the cause of death with the subjective and sometimes inaccurate medical records. Repeat revascularization was defined as a second percutaneous coronary intervention or redo CABG surgery to deal with graft failure or high-grade native coronary artery stenosis in the distal to the anastomosis.

Additionally, the values of LVEF and LVEDD prior to surgery, before discharge, and at the last follow-up in the severe group were also recorded.

### Statistical analysis

This study protocol was approved by the ethics committee of *Zhongshan* hospital *Fudan* University, and was consistent with the *Declaration of Helsinki*.

Categorical variables were represented as frequency distributions and single percentages. Values of continuous variables were expressed as a mean ± standard deviation (SD). Normally distributed continuous variables were compared using a *Student t-*test, non-normally distributed continuous variables using the *Mann-Whitney U* test, and categorical variables were compared by χ^2^ or *Fisher’s exact* test, where appropriate. Multiple logistic regression analysis was conducted to identify whether preoperative LVEF had independent influences on in-hospital clinical outcomes. Follow-up survival was conducted by Kaplan-Meier method with log-rank test for group comparisons. Potential independent risk factors for follow-up all-cause mortality were estimated by multiple *cox* regression analysis. All statistical tests were two-sided. Results were considered statistically significant at a level of *p* less than 0.05. All analyses were performed with the SPSS statistical package version 17.0 (SPSS Inc., Chicago, IL, USA).

## Results

### Study population

From January 2010 to December 2014, a total of 3274 patients received first, scheduled CABG surgery in our medical center. One thousand two hundred and forty-two patients were excluded due to concomitant left ventricle aneurysm, concomitant post-infarction ventricular septal defect, concomitant medium to severe mitral regurgitation or aortic regurgitation, concomitant acquired or congenital cardiac or aortic surgery, and use of cardiopulmonary bypass, leaving 2032 eligible patients for data analysis. According to preoperative LVEF, 128 patients with preoperative LVEF of 35% or less were entered into the severe group, 680 patients with preoperative LVEF of 36-50% into the impaired group, and the remaining 1224 patients with preoperative LVEF of more than 50% into the normal group.

Baseline characteristics of the cohort are shown in Table 1. Patients in the severe group compared to the other 2 groups were more likely to present with congestive heart failure and enlarged left ventricle. No significant differences were found between the severe group and the other 2 groups in the age and the proportion of older age, female, recent smoking, diabetes, hypertension, hyperlipemia, chronic obstructive pulmonary disease, renal dysfunction, prior cerebro-vascular accident, history of percutaneous coronary intervention, recent myocardial infarction, and coronary artery lesion.

**Table 1:** Baseline and procedure characteristics of the cohort

	Severe group (*n* = 128)	Impaired group (*n* = 680)	Normal group (*n* = 1224)	*p* value p_1_ **|** p_2_
LVEF (%)	31.0 ± 2.9	44.2 ± 3.6	58.9 ± 4.9	<0.001 **|** < 0.001
Age (years)	65.2 ± 8.2	66.6 ± 7.9	66.2 ± 7.1	0.07 **|** 0.14
Older age	75 (58.6%)	412 (60.6%)	709 (57.9%)	0.67 **|** 0.88
Female	27 (21.1%)	160 (23.5%)	271 (22.1%)	0.55 **|** 0.07
Recent smoking	21 (16.4%)	148 (21.8%)	236 (19.3%)	0.17 **|** 0.43
Diabetes mellitus	31 (24.2%)	218 (32.1%)	369 (30.1%)	0.08 **|** 0.16
Hypertension	73 (57.0%)	426 (62.6%)	760 (62.1%)	0.23 **|** 0.26
Hyperlipemia	19 (14.8%)	131 (19.3%)	211 (17.2%)	0.24 **|** 0.49
COPD	17 (13.3%)	75 (11.0%)	128 (10.5%)	0.46 **|** 0.33
Renal dysfunction	5 (3.9%)	41 (6.0%)	83 (6.8%)	0.34 **|** 0.21
Prior CVA	17 (13.3%)	108 (15.9%)	184 (15.0%)	0.46 **|** 0.60
History of PCI	24 (18.8%)	152 (22.4%)	285 (23.3%)	0.37 **|** 0.25
Recent MI	28 (21.9%)	131 (19.3%)	198 (16.2%)	0.50 **|** 0.10
Congestive heart failure	38 (29.7%)	115 (16.9%)	128 (10.5%)	0.001 **|** < 0.001
Coronary artery lesion				
Triple-vessel	116 (90.6%)	583 (85.7%)	1043(85.2%)	0.14 **|** 0.09
Left main	33 (25.8%)	196 (28.8%)	365 (29.8%)	0.48 **|** 0.34
Enlarged left ventricle	51 (39.8%)	98 (14.4%)	118 (9.6%)	<0.001 **|** < 0.001
Number of grafts	3.0 ± 0.6	3.1 ± 0.6	3.1 ± 0.7	0.08 **|** 0.12
Use of IMA	128 (100%)	674 (99.1%)	1216 (99.3%)	0.29 **|** 0.36
Use of SVG	115 (85.9%)	628 (92.4%)	1148 (93.8%)	0.34 **|** 0.09
Use of RA	21 (16.4%)	87 (12.8%)	136 (11.1%)	0.27 **|** 0.08
Urgent switch to on-pump	5 (3.9%)	11 (1.6%)	14 (1.1%)	0.09 **|** 0.01
Prophylactic use of IABP	18 (14.1%)	12 (1.8%)	0	<0.001 **|** < 0.001

p_1_, p value for the severe group vs. the impaired group; p_2,_ p value for the severe group vs. the normal group; LVEF, Left ventricular ejection fraction; COPD, chronic obstructive pulmonary disease; CVA, cerebro-vascular accident; PCI, percutaneous cardiac intervention; MI, myocardial infarction; IMA, internal mammary artery; SVG, saphenous vein graft; RA, radial artery; IABP, intra-aortic balloon pump

Procedure characteristics of the cohort are shown in Table 1. Patients in the 3 groups received a similar number of distal anastomoses and similar proportions of use of internal mammary artery and saphenous vein graft as well as radial artery. And, patients in the severe group compared to the other 2 groups were more likely to receive prophylactic use of IABP. In addition, patients in the severe group compared to the normal group were more likely to receive urgent switch from off-pump to on-pump CABG (3.9% vs. 1.1%, *p* = 0.028).

### In-hospital clinical outcomes

As shown in Table 2, patients in the severe group compared to the other 2 groups had higher incidences of postoperative low cardiac output and prolonged ventilation, longer length of ICU stay, and were more likely to receive IABP support on an “as needed” basis. No significant differences were found between the severe group and the impaired group and between the severe group and the normal group in the incidences of postoperative new onset of myocardial infarction, reoperation for bleeding, stroke, acute renal failure requiring hemodialysis and deep sternal wound infection, and the proportion of red blood cell transfusion.

**Table 2:** Clinical outcomes after surgery

	Severe group	Impaired group	Normal group	*P* value (p_1_ | p_2_)
In-hospital outcomes	n = 128	n = 680	n = 1224	
In-hospital mortality	6 (4.7%)	11 (1.6%)	14 (1.1%)	0.03 | 0.002
Circulatory morbidity				
New onset of MI	5 (3.9%)	17 (2.5%)	20 (1.6%)	0.37 | 0.07
Low cardiac output	25 (19.5%)	55 (8.1%)	50 (4.1%)	<0.001 | <0.001
IABP support	25 (19.5%)	55 (8.1%)	50 (4.1%)	<0.001 | <0.001
Reoperation for bleeding	3 (2.3%)	11 (1.6%)	15 (1.2%)	0.56 | 0.29
Prolonged ventilation (>48 h)	11 (8.6%)	24 (3.5%)	13 (1.1%)	0.01 | <0.001
Stroke	5 (3.9%)	25 (3.7%)	41 (3.3%)	0.90 | 0.74
Acute renal failure	4 (3.1%)	10 (1.5%)	15 (1.2%)	0.19 | 0.08
DSWI	5 (3.9%)	13 (1.9%)	20 (1.6%)	0.16 | 0.07
Red blood cell transfusion	24 (18.8%)	89 (13.1%)	165 (13.5%)	0.09 | 0.10
Length of ICU stay (d)	3.9 ± 1.9	1.9 ± 1.2	1.6 ± 1.1	<0.001 | <0.001
Follow-up outcomes	n = 122	*n* = 610	*n* = 1103	
All-cause mortality	11 (9.0%)	45 (7.4%)	73 (6.6%)	0.53 | 0.32
Cardiac-cause deaths	4 (4.3%)	11 (1.8%)	16 (1.5%)	0.29 | 0.13
Repeat revascularization	4 (3.3%)	14 (2.3%)	26 (2.4%)	0.52 | 0.53

p_1_, p value for the severe group vs. the impaired group; p_2,_ *p* value for the severe group vs. the normal group; MI, myocardial infarction; IABP, intra-aortic balloon pump; DSWI, deep sternal wound infection; ICU, intensive care unit

A total of 31 patients died during the same hospitalization or within 30 days of operation, and the in-hospital mortality was 1.5%. The in-hospital mortality in the severe group was significantly higher than that in the impaired group (4.7% vs. 1.6%, *p* = 0.039) and that in the normal group (4.7% vs. 1.1%, *p* = 0.008). The causes of death are listed in Table 3. The leading causes of death were infection and postoperative low cardiac output.

**Table 3:** Causes of death in the cohort

	Normal group	Impaired group	Severe group
In-hospital			
No. patients	14	11	6
Infection	7	4	2
Low cardiac output	3	3	2
Malignant arrhythmia	2	1	1
Gastrointestinal bleeding	2	2	1
Other	0	1	1
Follow-up			
No. patients	73	45	11
Infection	34	22	3
Heart failure	12	8	3
Myocardial infarction	4	3	1
Cancer	9	4	2
Sudden death	4	2	0
Pulmonary failure	4	2	1
Hepatic failure	2	2	0
Stroke	2	1	1
Other	2	1	0

The impacts of grouping (the impaired group vs. the severe group, and the normal group vs. the severe group) on in-hospital death and major postoperative morbidity after adjustment for potential confounders (age, gender, recent smoking, diabetes, hypertension, hyperlipaemia, chronic obstructive pulmonary disease, renal dysfunction, prior cerebro-vascular accident, history of percutaneous cardiac intervention, history of myocardial infarction, triple-vessel coronary artery disease, left main coronary artery disease, and the number of distal anastomoses) are shown in Table 4. Multivariate logistic regression analysis showed that grouping had independent influences on the development of in-hospital mortality (OR = 0.27, 95%CI 0.10-0.67, *p* = 0.008 for the normal group vs. the severe group, and OR = 0.38, 95%CI 0.18-0.92, *p* = 0.041 for the impaired group vs. the severe group), postoperative low cardiac output (OR = 0.21, 95% CI 0.16-0.35, *p* < 0.001 for the normal group vs. the severe group, and OR = 0.39, 95% CI 0.26-0.71, *p* = 0.002 for the impaired group vs. the severe group), and prolonged ventilation (OR = 0.17, 95% CI 0.09-0.31, *p* = 0.001 for the normal group vs. the severe group, and OR = 0.41, 95%CI 0.20-0.88, *p* = 0.032 for the impaired group vs. the severe group).

**Table 4:** Risk-adjusted effect of grouping on in-hospital outcomes

Events	Severe group	Impaired group OR (95%CI), p value	Normal group OR (95%CI), *p* value
In-hospital mortality	1.0	0.38 (0.18-0.92), *p* = 0.04	0.27 (0.10-0.67), *p* = 0.008
New onset of MI	1.0	0.66 (0.28-1.87), *p* = 0.38	0.45 (0.18-1.39), *p* = 0.08
Low cardiac output	1.0	0.39 (0.26-0.71), *p* = 0.002	0.21 (0.16-0.35), *p* < 0.001
Reoperation for bleeding	1.0	0.71 (0.23-2.62), *p* = 0.48	0.55 (0.18-1.92), *p* = 0.24
Prolonged ventilation	1.0	0.41 (0.20-0.88), *p* = 0.03	0.17 (0.09-0.31), *p* = 0.001
Stroke	1.0	0.96 (0.41-2.76), *p* = 0.84	0.87 (0.38-2.36), *p* = 0.81
Acute renal failure	1.0	0.51 (0.21-1.67), *p* = 0.29	0.42 (0.18-1.35), *p* = 0.09
DSWI	1.0	0.52 (0.21-1.75), *p* = 0.19	0.42 (0.17-1.36), *p* = 0.09
Red blood cell transfusion	1.0	0.67 (0.42-1.21), *p* = 0.11	0.69 (0.44-1.28), *p* = 0.14

MI, myocardial infarction; DSWI, deep sternal wound infection

Adjusted confounders included age, gender, recent smoking, diabetes, hypertension, hyperlipaemia, chronic obstructive pulmonary disease, renal dysfunction, prior cerebro-vascular accident, history of percutaneous cardiac intervention, history of myocardial infarction, congestive heart failure, triple-vessel coronary artery disease, left main coronary artery disease, and number of grafts

### Follow-up clinical outcomes

**Fig. 1:**
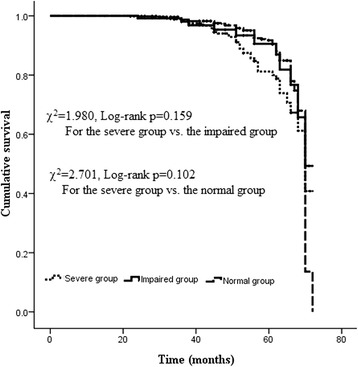
Actuarial curves of mid-term survival after OPCAB surgery

A total of 1835 patients (122 patients in the severe group, 610 patients in the impaired group, and 1103 patients in the normal group), accounting for 90.3% of all included patients, received follow-up lasting 46.2 ± 10.1 months (22-72 months). One hundred and twenty-nine patients died during follow-up, and the mid-term survival was 93.0%. No significant differences were found in either all-cause mortality or cardiac-cause mortality between the severe group and the impaired group and between the severe group and the normal group. As shown in Table 3, the main causes of death were infection and heart failure. As shown in Fig. 1, Kaplan-Meier curves showed a similar cumulative follow-up survival between the severe group and the impaired group (χ^2^ = 1.980, Log-rank *p* = 0.159) and between the severe group and the normal group (χ^2^ = 2.701, Log-rank *p* = 0.102). Multivariate *Cox* regression analysis showed that grouping (the severe group vs. the impaired group, and the severe group vs. the normal group) was not an independent predictors of follow-up all-cause mortality (HR = 1.32, 95% CI 0.78-2.48, *p* = 0.612; HR = 1.18, 95% CI 0.81-2.15, *p* = 0.453, respectively).

A total of 44 patients (26 patients in the normal group, 14 patients in the impaired group, and 4 patients in the severe group) received repeat revascularization during the follow-up period of 46.2 ± 10.1 months. No significant difference was found in the rate of repeat revascularization between the severe group and the other 2 groups. Only 2 patients underwent redo-CABG because of occlusion of the left internal mammary artery bypass grafting to the left anterior descending artery, and the remaining 42 patients underwent further percutaneous coronary intervention.

### LVEF and LVEDD

**Fig. 2:**
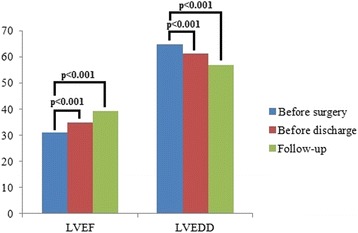
Changes in left ventricular ejection fraction and endo-diastolic diameter with time going. LVEF, left ventricular ejection fraction; LVEDD, left ventricular end-diastolic diameter

As shown in Fig. 2, mean LVEF values, as measured before surgery, before discharge and at the last follow-up, significantly improved from 31.0 ± 2.9% to 34.9 ± 3.2% before discharge (*p* < 0.001), and to 39.2 ± 5.3% at the last follow-up (*p* < 0.001). Mean LVEDD significantly decreased from 64.7 ± 5.8 mm before surgery to 61.2 ± 6.5 mm before discharge (*p* < 0.001), and to 56.8 ± 5.7 mm at the last follow-up (*p* < 0.001).

## Discussion

The important finding of this study was that patients with preoperative severe left ventricular dysfunction compared to preoperative mild to moderate left ventricular dysfunction and preoperative normal left ventricular function increased the risk of in-hospital death and the incidences of postoperative low cardiac output and prolonged ventilation after receiving surgical revascularization. In this study, baseline characteristics of the 3 groups were similar, except LVEF, and the proportions of congestive heart failure and enlarged left ventricle. The in-hospital mortality in the severe group was 4.7%, which was similar to that described previously (2.2-6.8%) [3, 6–8, 10, 14]. This study showed an increased risk of in-hospital death and increased incidences of postoperative low cardiac output and prolonged ventilation with the severe group compared to the impaired group and the normal group in univariate factor analysis and then multivariate logistic regression analysis. And, patients in the severe group compared to the other 2 groups had higher proportions of IABP support on an “as needed” basis and prolonged ICU stay. These results suggested that preoperative severe left ventricular dysfunction, together with surgical trauma, increased the incidences of postoperative low cardiac output and prolonged ventilation, and thus increasing the rate of IABP support on an “as needed” basis and the length of ICU stay, all of which may contribute to the increase of in-hospital mortality in patients with coronary artery disease and severe preoperative left ventricular dysfunction who underwent surgical revascularization. Previously, Shapira and colleagues [11] conducted a study including 115 patients with LVEF of less than 30% undergoing isolated CABG vs. 2335 patients with LVEF of more than 30% receiving isolated CABG, and found that patients with preoperative LVEF of less than 30% compared to preoperative LVEF of more than 30% presented slightly higher operative mortality and significantly higher morbidity (higher incidences of respiratory, renal, and vascular complications). This evidence was in line with the results of the current study.

Another important finding of this study was that patients between the severe group and the other 2 groups received similar mid-term clinical outcomes in terms of mid-term all-cause mortality and repeat revascularization. This study reported 9.0% all-cause mortality for patients with coronary artery disease and preoperative severe left ventricular dysfunction who received surgical revascularization, which was lower than the results of a previous study conducted by Yoo and colleagues [5]. The reason for this difference may be the duration of follow-up, regarding that median follow-up time was 46.2 months in the current study, and may be the study population, regarding that the current study only included patients undergoing isolated OPCAB surgery, whereas Yoo’s study included 476 CABG patients undergoing concomitant mitral surgery and surgical ventricular reconstruction. In this study, Kaplan-Meier curves displayed a similar cumulative follow-up survival between the severe group and the other 2 groups, and multivariate Cox regression indicated that grouping was not a significant variable related to the mid-term all-cause mortality, both of which suggested that patients in the severe group shared a similar mid-term all-cause mortality with patients in the other 2 groups. This evidence agreed with the outcomes of Davoodi’s study [6], but was differed from the results of another study [7]. The reason for this difference may be the duration of follow-up, regarding that median follow-up time was 46.2 months in the current study, and may be the definition of severe left ventricular dysfunction, regarding that the current study defined severe left ventricular dysfunction as LVEF of 35% or less. In addition, this study showed that the incidence of repeat revascularization in the severe group was 3.3%, which was similar to that described in previous studies [13, 15]. No significant difference was found in the rate of repeat revascularization between the severe group and the other 2 groups, suggesting there may be no causality between preoperative left ventricular function and repeat revascularization after OPCAB surgery.

This study also found that for patients in the severe group, mean LVEF improved gradually and mean LVEDD decreased gradually after surgery, which suggested that patients with coronary artery disease and preoperative severe left ventricular dysfunction benefited from surgical revascularization. This evidence was in line with the results of previous reports [15, 16].

In this study, the severe group compared to the other 2 groups had significantly higher proportions of congestive heart failure and EuroSCORE >6. The reason for this difference may be related to preoperative LVEF. The severe group compared to the normal group had a significantly higher incidence of urgent switch from off-pump to on-pump CABG, which suggested that patients with preoperative severe left ventricular dysfunction undergoing off-pump CABG had a much higher exposure to the risks. This study also showed that the severe group compared to the other 2 groups had a significantly higher proportion of prophylactic use of IABP support. With the aid of IABP support, especially prophylactic use before surgery, patients with coronary artery disease and severe left ventricular dysfunction who received OPCAB surgery were associated with acceptable in-hospital mortality. This evidence agreed with the outcomes of a previous study [21].

Dual anti-platelet therapy post-CABG was routinely used in our medical. The evidence supporting routine dual anti-platelet therapy post-CABG for the prevention of clinical events is based primarily on nonrandomized evidence with substantial inherent limitations, and the small randomized studies published to date have only demonstrated improved graft patency [19]. Nonetheless, some clinical practice guidelines recommend 1 year of dual anti-platelet therapy for acute coronary syndrome after CABG. Potential benefits of dual anti-platelet therapy in patients undergoing CABG are derived from stabilization of existing plaque, improving vein graft patency, and continued protection of existing stents [22]. In addition, dual anti-platelet therapy can theoretically: 1) reduce the risk of acute coronary syndrome recidivism in non-culprit arteries irrespective of bypass grafting; 2) augment platelet inhibition in aspirin non-responders; 3) reduce the risks of associated non-coronary conditions (e.g., stroke in patients not receiving anticoagulation for atrial fibrillation); and 4) mediate improved outcomes through non-platelet receptor interactions (e.g., decreased infarct size via ticagrelor erythrocyte adenosine reuptake inhibition) [23].

There are several limitations of this study. First, this study was a single center, retrospective, clinical observational study that involved a small sample size (only 128 Chinese patients were included in the severe group), which may have influenced the generalizability of the results. A final determination would need multi-center and multinational studies involving larger sample sizes. Second, LVEF, as an index of left ventricular function, was calculated based on left ventricular end-diastolic volume and left ventricular end-systolic volume estimates, which might fluctuate, particularly in patients with unstable hemodynamics and various medical therapies. Finally, the duration of follow-up was relatively short. The long-term outcomes need a further observation.

## Conclusion

This single center retrospective study found that patients with severe left ventricular dysfunction compared to mild to moderate left ventricular dysfunction and normal left ventricular function increased the risk of in-hospital death and incidences of low cardiac output and prolonged ventilation, but shared similar mid-term clinical outcomes in terms of all-cause mortality and repeat revascularization after OPCAB surgery.

## Abbreviations

CABG: Coronary artery bypass grafting surgery
IABP: Intra-aortic balloon pump
ICU: Intensive care unit
LVEDD: Left ventricular end-diastolic diameter
LVEDV: Left ventricular end-diastolic volume
LVEF: Left ventricular ejection fraction
LVESV: Left ventricular end-systolic volume
OPCAB: Off-pump coronary artery bypass grafting surgery
